# The Evolving Roles of Nuclear Cardiology

**DOI:** 10.2174/157340309787048112

**Published:** 2009-01

**Authors:** Andrea De Lorenzo

**Affiliations:** Instituto Nacional de Cardiologia, Rio de Janeiro, Brazil

**Keywords:** Myocardial perfusion imaging, single-photon emission computed tomography, positron emission tomography.

## Abstract

The use of cardiac imaging modalities has grown steadily, and cardiac nuclear studies constitute a large part of this number. Nuclear Cardiology is often mistakenly considered a synonym of myocardial perfusion imaging (MPI), but has broader applications, including metabolic imaging, innervation imaging, among other technologies. MPI has been a powerful diagnostic and prognostic tool in the assessment of patients for known or suspected CAD for decades, and is now increasingly used for the evaluation of the anti-ischemic effects of various therapies, according to changes in left ventricular perfusion defect size defined by sequential MPI. Neuronal dysfunction identified with iodine-123-metaiodobenzylguanidine may give information on prognosis in different disease conditions, such as after myocardial infarction, in diabetes and dilated cardiomyopathy. Molecular imaging may identify the predominant cellular population in the atherosclerotic plaque and help predict the likelihood of clinical events. Therefore, although its usefulness is well established, Nuclear Cardiology remains a moving science, whose roles keep in pace with evolving clinical needs and expectations.

In association with the elevated prevalence, morbidity and mortality of coronary artery disease (CAD) worldwide, there has been a steady increase in the use of cardiac imaging techniques, including those based on Nuclear Medicine. In fact, cardiac studies have outgrown Nuclear Medicine and have constituted an individual subspecialty, Nuclear Cardiology. This imaging modality is often, although incorrectly, considered as a synonym for myocardial perfusion imaging (MPI), which by its turn means the evaluation of myocardial perfusion after stress versus at rest, using a variety of tracers and stressor agents in different protocols. It extends far beyond, though, including metabolic imaging, innervation imaging, among other technologies. This review will discuss current and evolving applications of Nuclear Cardiology, starting with MPI, which remains its cornerstone.

## MYOCARDIAL PERFUSION IMAGING

For the last 2 decades, MPI has been an essential part of the diagnostic and prognostic assessment of patients for known or suspected CAD. However, as time went by and new demands and technologies have been developed, other applications of MPI have emerged, and its roles have advanced continuously. In the 1980’s, planar and then tomographic (single photon emission computed tomography, SPECT) perfusion imaging was found to reliably diagnose CAD [1,2]. In the 1990’s the prognostic value of MPI was defined [3-5]. Later, with the advent of gating, left ventricular ejection fraction could be obtained, thus providing additional diagnostic and prognostic information [6,7]. 

### Diagnostic Assessment

MPI is a useful tool for the diagnosis of CAD [1,2]. It is most effective when used in patients with an intermediate pretest likelihood of CAD, since if the likelihood is low, a perfusion abnormality may be a false-positive finding, while in those with a high pretest likelihood of CAD a normal MPI study may be a false-negative for the presence of angiographically significant coronary obstruction (i.e., coronary stenoses > 50% of luminal diameter) and the test may not able to exclude the presence of CAD. Nonetheless, even in patients with high likelihood of CAD, MPI can still be used for risk stratification [8]. In fact, the principles regarding diagnostic applications of MPI have not changed over the years, in contrast to the prognostic use of MPI, which has grown significantly.

### Prognostic Assessment

MPI may be applied for prognostic evaluation both for patients with known or suspected CAD. It relies on 2 principles: first, a normal MPI study confers a low (<1%) short-term risk of cardiac events [9]; second, there is a relationship between myocardial ischemia and cardiac events such that the risk of events increases exponentially as ischemia increases. Even in patients with a high likelihood of CAD or definite CAD, a normal MPI defines those who have a < 1% risk of cardiac events in the next 1-2 years. This usually defines a group in which conservative management (i.e., avoiding myocardial revascularization) is appropriate [10]. When some conditions are present, like diabetes, inability to exercise leading to the use of pharmacologic stress, abnormal rest ECG or exercise-induced ST segment depression, a normal MPI study still delineates low risk, but somewhat increased compared with patients with normal MPI and none of those [11,12]. For patients with abnormal studies, the risk of events is associated with the magnitude of ischemia. The latter is the strongest determinant of the referral to coronary angiography and also the strongest predictor of benefit with revascularization [13]. Besides that, a number of other findings obtained from MPI studies, such as abnormal lung uptake or transient left ventricular dilatation, have consistently been associated with increased risk [14, 15].

### Therapeutic Assessment/MPI as a “Gatekeeper” 

An increasingly used application of Nuclear Cardiology is to evaluate the anti-ischemic effects of various therapies by means of identifying changes in left ventricular perfusion defect size by sequential MPI and thereby track patient risk for subsequent cardiac events on the basis of the magnitude of ischemia suppression. An example is the COURAGE (Clinical Outcomes Utilizing Revascularization and Aggressive Drug Evaluation) trial [16], which evaluated percutaneous coronary intervention (PCI) plus optimal medical therapy (OMT) *vs.* OMT alone in stable CAD patients. A subgroup of the total population had MPI performed before and after treatment (mean 1 year after enrollment), and in this nuclear substudy, the results showed that ischemia reduction was greater with PCI+OMT than with OMT alone (33.3% versus 19.8% had ≥5% reduction of ischemia). For all patients combined, the death or myocardial infarction rate was 13.4% for patients who had ≥5% reduction of ischemia and 24.7% for those who did not have reduction at the follow-up study. This study indicated that, for stable CAD patients, having knowledge of the ischemic burden is helpful in decision-making with respect to selecting initial therapy, and regardless of the therapeutic modality used, assessment of the response to therapy, which is related to long-term outcome, is also important. Another aspect of this same application of MPI is the identification of revascularization candidates according to the amount of jeopardized myocardium and the survival benefit that a patient may obtain from a specific therapy as a function of MPI results. MPI has been able to discriminate (as a “gatekeeper”) which patients will likely benefit from invasive coronary angiography followed by coronary revascularization and who may be well suited with medical therapy [13]. Quantitative analysis of MPI studies has been important in this regard, since this kind of analysis relies on the demonstration of a threshold value of ischemia which will lead to each type of management. 

### Screening Asymptomatic Populations for CAD

The noninvasive detection of silent CAD is an important clinical goal in certain high-risk asymptomatic populations. Occult CAD precedes the onset of clinically manifest CAD, which too often presents as sudden death or acute myocardial infarction. Detection of occult CAD in a preclinical stage might lead to risk factor modification and institution of treatment, which would prevent or delay the onset of clinical disease, since there is now good evidence that aggressive risk factor modification can slow the progression of atherosclerosis. Bayesian analysis indicates that the detection of occult CAD by noninvasive testing should be more accurate in asymptomatic groups with high CAD prevalence, since test performance is worse in low prevalence populations [17]. As a result, screening is not considered appropriate for the general population, given the sensitivity and specificity of even the best noninvasive test. Screening seems more suitable for selected, high risk groups, such as patients with chronic renal failure, metabolic syndrome, and diabetics; in the latter, several studies have demonstrated a high prevalence of silent CAD detected with MPI [18, 19]. 

### Myocardial Viability

Noninvasive imaging to determine the presence and extent of dysfunctional but viable myocardium has become an important component of the diagnostic assessment of patients with CAD and depressed left ventricular function. It is now well established that left ventricular dysfunction in patients with CAD is not always an irreversible process related to previous myocardial infarction because left ventricular function may improve substantially after revascularization procedures in patients with chronic CAD, and this may translate into an improvement in survival [20]. The differentiation of viable from nonviable myocardium is highly relevant in patients with left ventricular dysfunction considered for revascularization, because these procedures often have high morbidity and mortality rates in this subset of patients, even though this is the population that ultimately benefits most from revascularization. Therefore, accurate methods to detect viable myocardium are essential to select the patients for whom the risks are justified. Nuclear Cardiology techniques to assess viability have evolved tremendously in recent years. In SPECT, there are various imaging protocols, the most frequently used being the thallium-201 protocols (rest/redistribution and reinjection studies) [21, 22]. According to the hypothesis of resting hypoperfusion (hibernation), rest images demonstrate thallium uptake early after injection, which represent regional myocardial blood flow, whereas delayed uptake reflects cell membrane integrity. Since 3-4 hour redistri-bution images may underestimate the presence of viable myocardium, reinjection of thallium at rest after redis-tribution imaging may improve the assessment of myocardial ischemia and viability in up to 49% of apparently irreversible defects [22]. As technetium-99m tracers are widely used due to favorable imaging properties compared to thallium, the use of these tracers for the assessment of viability has been studied, and quantitative measures of regional technetium-99m sestamibi activity have been shown to correlate with estimates of viability with other techniques [23]. Glucose metabolism assessment with F-18 fluorodeoxi-glucose (FDG) positron emission tomography (PET) also allows the identification of viable myocardium, with higher sensitivity; a perfusion-metabolism mismatch (reduced blood flow associated with preserved or enhanced FDG uptake) identifies potentially reversible myocardial dysfunction [24].

## MYOCARDIAL INNERVATION IMAGING

Iodine-123-metaiodobenzylguanidine (MIBG) was first reported as a potential neuronal imaging agent for detection of pheocromocytoma, since it has structural similarity with norepinephrine and therefore is taken up by peripheral sympathetic nerves. Further studies have shown reduced localization in denervated regions of the myocardium, after myocardial infarction, in diabetes and dilated cardiomyopathy, among other conditions [25, 26]. MIBG imaging evaluates the sympathetic nervous system and has been shown to be an important prognosticator in patients with heart failure [27].

## POSITRON EMISSION TOMOGRAPHY (PET)

The lack of widespread availability of PET cameras and radiotracers, their high costs and reimbursement issues have limited the clinical use of this technology. Recent developments are likely to change this scenario, though, since there has been a growth in the number of PET cameras and rubidium-82 generators used for perfusion imaging. This leads to real benefit, since positron emission tomography (PET) has several technical advantages over SPECT, including higher spatial resolution, accurate attenuation correction, higher temporal resolution and quantitative imaging capability that afford measurement of rapidly changing radiotracer activity concentrations in blood and myocardium. All of those determine a clinical advantage over SPECT, which often uncovers only the territory supplied by the most severe stenosis and therefore may underestimate the significance of coronary lesions; in this regard, PET has superior capability to detect multivessel CAD [28,29]. 

## THE ASSOCIATION WITH COMPUTED TOMOGRAPHY: SPECT-CT/ PET-CT

Computed tomography of the coronary arteries (CTA) has gained enormous popularity in a short time period, and has been taken by some as a “threat” for Nuclear Cardiology. CTA and MPI differ in what each evaluates- anatomy for one and physiology for the other. Hybrid systems are now available allowing the combined assessment of anatomy and function, linking the anatomic richness of CTA and the functional importance of perfusion either from PET or SPECT. MPI and CTA are most likely complementary imaging modalities, with the choice and sequence of testing depending on the particular clinical question and specific patient population being evaluated [30, 31]. 

## FATTY ACID IMAGING

BMIPP is a fatty acid analog which allows metabolic imaging since most of the energy requirement of the normal myocardium under aerobic status is derived from the metabolism of fatty acids. Since under ischemia or in heart failure, beta-oxidation of fatty acids in mitochondria is reduced, early washout of BMIPP from the myocardium occurs and the uptake detected by SPECT is reduced. BMIPP imaging may be used in unstable angina, vasospastic angina and cardiomyopathies, among other indications [32,33]. In particular, BMIPP metabolic imaging may be used to reveal “ischemic memory”, since even after resolution of chest pain, metabolic abnormalities may persist, and BMIPP images may be abnormal, with sensitivity of ~70-80% and specificity of ~90% [34].

## MOLECULAR IMAGING/PLAQUE IMAGING

The management of CAD has almost always been based on demonstration of the severity of luminal stenosis; such an approach, however, does not characterize the plaque morphology that happens to be the major determinant of clinical outcome [35,36]. Appropriate targeting strategies with radionuclide imaging techniques may identify the predominant cellular population in the atherosclerotic plaque and help predict the likelihood of clinical events. It is now well recognized that progressive luminal stenosis of the coronary artery is generally not associated with an acute event and that thrombotic occlusion usually occurs as a result of plaque rupture [37, 38]. The plaques that are vulnerable to rupture have large lipid cores, attenuated fibrous cap and intense infiltration of macrophages, which release metalloproteinases that digest matrix and induce fibrous cap rupture. Plaque rupture exposes thrombogenic lipid core leading to thrombotic luminal obstruction. Plaque imaging in Nuclear Cardiology has targeted most often macrophages or lipid cores. However, the uptake of radiolabeled antibodies or cells in the atherosclerotic lesion is a small fraction of the injected dose, what makes the background radioactivity a major contributor to the image and decreases the contrast between lesion and nonlesion tissue. The difficulty in identifying lesions *in vivo* is a problem and highlights the difficulty in the use of this innovative technique. In the carotids, F-18 FDG has been studied for plaque inflammation and technetium-99m-annexin SPECT has been evaluated for apoptosis within the plaque, both known markers of plaque instability [39,40]. 

## CONCLUSION

Although initially applied for diagnostic purposes, Nuclear Cardiology has grown and keeps moving. Many advances have increased its applications, and many more, regarding novel instrumentation techniques and new tracers are on their way to clinical practice. Perhaps the greatest potential for the future of Nuclear Cardiology lies in molecular imaging, and Nuclear Cardiology may hopefully achieve one of the most difficult goals in Cardiology: to identify the vulnerable, rupture-prone coronary plaque. 
